# Dormancy in the origin, evolution and persistence of life on Earth

**DOI:** 10.1098/rspb.2024.2035

**Published:** 2025-01-08

**Authors:** Kevin D. Webster, Jay T. Lennon

**Affiliations:** ^1^ Diné College, Tsaile, AZ, USA; ^2^ Planetary Science Institute, Tucson, AZ, USA; ^3^ Indiana University, Bloomington, IN, USA

**Keywords:** biodiversity, astrobiology, seed bank, prebiotic chemistry, extinction

## Abstract

Life has existed on Earth for most of the planet’s history, yet major gaps and unresolved questions remain about how it first arose and persisted. Early Earth posed numerous challenges for life, including harsh and fluctuating environments. Today, many organisms cope with such conditions by entering a reversible state of reduced metabolic activity, a phenomenon known as dormancy. This process protects inactive individuals and minimizes the risk of extinction by preserving information that stabilizes life-system dynamics. Here, we develop a framework for understanding dormancy on early Earth, beginning with a primer on dormancy theory and its core criteria. We hypothesize that dormancy-like mechanisms acting on chemical precursors in a prebiotic world may have facilitated the origin of life. Drawing on evidence from phylogenetic reconstructions and the fossil record, we demonstrate that dormancy is prevalent across the tree of life and throughout deep time. These observations lead us to consider how dormancy might have shaped nascent living systems by buffering stochastic processes in small populations, protecting against large-scale planetary disturbances, aiding dispersal in patchy landscapes and facilitating adaptive radiations. Given that dormancy is a fundamental and easily evolved property on Earth, it is also likely to be a feature of life elsewhere in the universe.

## 1. Introduction

Life on Earth today can only originate from existing organisms, but there was a time in the distant past when life arose from non-living substances. During that period, before organisms diversified and transformed the planet, primitive life had to overcome several major obstacles. First, chemical building blocks needed to be supplied at rates that exceeded decay caused by processes such as hydrolysis, photolysis and thermal decomposition. Hydrothermal vents have been proposed as locations on early Earth where fluids with contrasting redox potentials interacted over sustained periods, leading to the generation of reduced substrates capable of directing essential life processes [1–3]. Second, primitive life required compartmentalization between protocell components and the external environment. The rise of membrane-bound vesicles made of fatty acids or phospholipids afforded protection and selective permeability, facilitating homeostasis and the establishment of proton gradients necessary for more sophisticated forms of metabolism [4]. Third, the emergence of life was susceptible to chance events and errors. The evolution of information-directed replication ensured that the blueprints for life were transmitted to offspring with sufficient fidelity, meeting the criteria for Darwinian evolution [5].

To persist through time, primitive organisms also had to contend with the forces of entropy. Individuals had to endure harsh and unpredictable conditions long enough to reproduce and expand into new, uninhabited landscapes, all without the evolutionary refinements that developed over the eons that followed. These problems could have been overcome in several non-mutually exclusive ways. For example, individuals could have survived by living in spatially restricted patches of habitat with relatively stable conditions that were suitable for growth and reproduction [6]. Those who venturing into less optimal habitats may have survived by living in groups, where protection was provided through cooperation, exchange of metabolites and division of labour [7]. Others exposed to more stressful conditions would have faced strong selective pressure, potentially leading to the rapid evolution of specialized traits that increased population stability [6].

**Figure 1.**
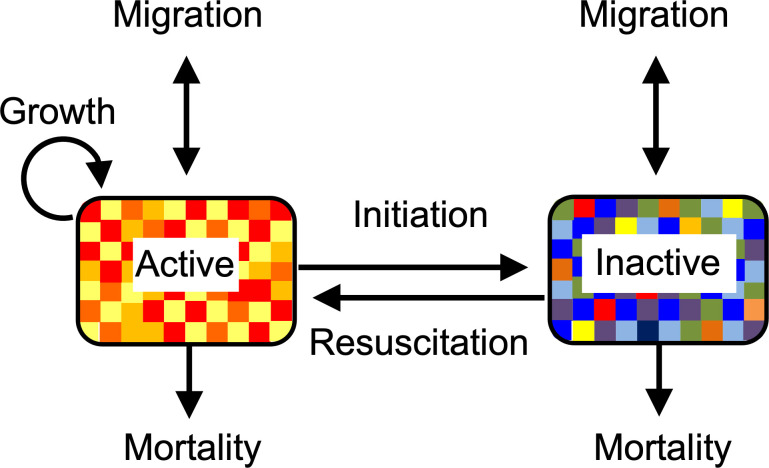
A generalized model of dormancy. Dormancy is a set of processes acting on agents that exist in different states of activity. Active agents transition into inactive agents through the process of dormancy initiation, while inactive agents transition into active states through the process of resuscitation. Transitions can occur stochastically or in a responsive manner. Mortality rates are assumed to be higher for active agents than for inactive agents, which can facilitate migration and colonization. This form of protection can lead to the accumulation of inactive individuals and the creation of a ’seed bank', which serves as a reservoir of information contained in agents (colours) that can generate complex behaviours through the preservation of diversity and memory. Modified figure from [10].

Dormancy is another strategy that probably played a crucial and multifaceted role in the persistence of life on early Earth. The ability of organisms to enter a reversible state of reduced metabolic activity could have contributed to the emergence of life in several ways. Beyond supporting higher abundances and greater biomass, dormancy would have maintained community-wide biodiversity, thereby ensuring sustained ecological functions, energy dissipation and material recycling [8]. By decreasing rates of mortality under suboptimal conditions, dormancy would have reduced the probability of local and global extinction events [9]. Also, dormancy creates a ‘seed bank’ of inactive individuals [10] (figure 1). The resuscitation of inactive propagules from this reservoir may have eliminated the need to repeatedly restart the life-building process on early Earth.

While dormancy has been invoked in the origin of life [11,12], its logical foundation and supporting evidence have yet to be rigorously developed. Here, we establish a framework to explore dormancy’s role in the emergence of life on Earth. We hypothesize that in a prebiotic world, dormancy-like mechanisms acting upon chemical precursors facilitated the origin of life. Drawing from phylogenetic reconstructions and evidence from fossil records, we trace the roots and potential origins of dormancy. We also examine how dormancy could have influenced population genetic processes, provided protection against planetary-scale disturbances, and ultimately contributed to the dispersion and diversification of life on Earth. Finally, given that dormancy is an easy-to-evolve property of living systems, we propose that it is likely to be a feature of life elsewhere in the universe.

## 2. A dormancy primer

Although initially developed to understand population dynamics in biology, the principles of dormancy can be applied to other systems, including cancer, network science and interacting particle systems [10]. In an abstract sense, dormancy can operate on any agent—defined as an individual component within a complex system that interacts with other agents and its environment—provided it meets the following criteria: (i) an agent can exist in different states of activity, (ii) an agent can transition between these states of activity and (iii) an agent experiences some degree of protection from decay while in a less active state. For simplicity, we consider an agent existing in one of two states of activity—‘on’ or ‘off’ (figure 1)—although, in some systems, agents can occupy states along a gradient or spectrum of activity and protection [13].

Dormancy provides benefits to agents in fluctuating environments [14]. At the individual level, dormancy reduces the probability of mortality under adverse conditions and allows an agent to resume activity when favourable conditions return. At the population level, dormancy minimizes variance in abundance over time, reducing the probability of extinction and thereby increasing geometric mean growth (i.e. fitness) [15]. The benefits of dormancy depend not only on the mechanisms by which agents transition between states of activity but also on the environmental dynamics in which they exist. For example, in a rapidly and unpredictably fluctuating environment, it may be optimal to transition between states in a stochastic manner consistent with bet-hedging [15]. However, in an environment that changes slowly or more predictably, it may be optimal to responsively transition between states of activity based on the interpretation of internal or external cues [14] (figure 1).

Regardless of the transitioning mechanism, dormancy results in the accumulation of inactive agents, forming what is known as a ‘seed bank’. This reservoir preserves information and diversity in the population of agents, which leads to emergent phenomena and complex dynamics [10]. There are a number of important attributes that affect seed bank behaviour. The absolute and relative sizes of the seed bank are critical for determining how dormancy influences population dynamics [10]. The turnover rate of the seed bank is also crucial and is influenced by the balance of processes governing transitions into and out of dormancy. Some inactive agents are short-lived, while others may persist in the seed bank for extended periods [9]. Physical and spatial aspects of the seed bank are important considerations. In some systems, inactive and active agents may be well-mixed. However, in other systems, movement is affected by differences in the size and density of active and inactive agents, resulting in spatially segregated seed banks (figure 1) [10]. Taken together, the features and attributes of dormancy can lead to rich and complex dynamics that are important for both living and non-living systems.

## 3. Dormancy in a prebiotic world

We propose that dormancy theory provides a valuable framework for understanding the dynamics of biomolecules both in contemporary living systems and under the prebiotic conditions of early Earth. We hypothesize that chemical dormancy may have played a crucial yet previously overlooked role in the origins of life. Of course, it is challenging to make unequivocal claims about how molecules behaved more than 4 billion years ago (Ba), prior to the emergence of cellular life. However, if we assume that the fundamental laws of physics and chemistry have remained consistent over time, observations of contemporary molecular dormancy could provide valuable insights into the processes that shaped prebiotic Earth.

### (a) Criteria for chemical dormancy

**Figure 2.**
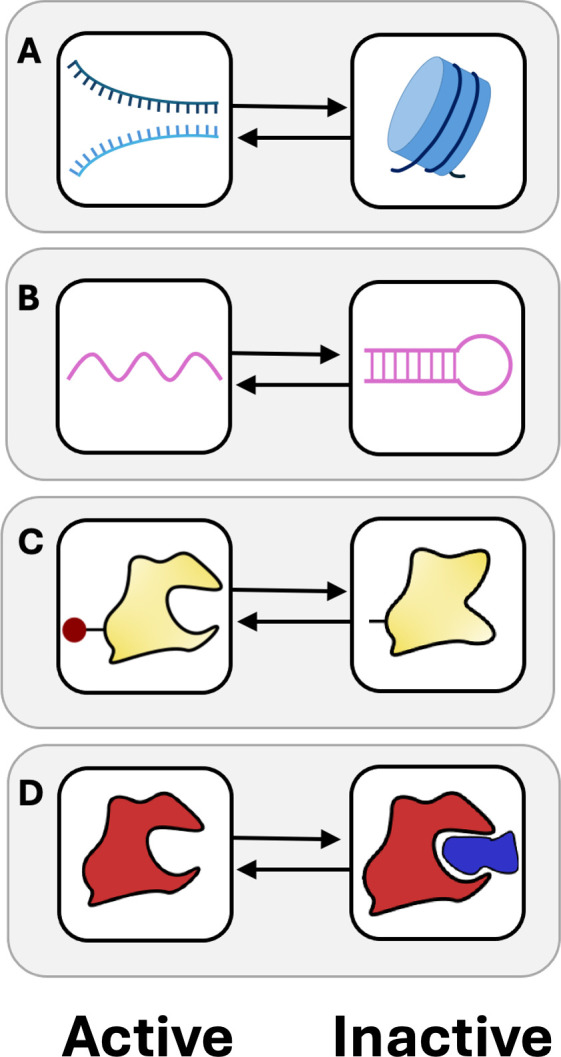
Examples of chemical dormancy. Biomolecules can be viewed as agents that exist in either active (left column) or inactive (right column) states. (A) In eukaryotic organisms, DNA typically exists in an inactive state, tightly bound to histones forming a nucleosome. DNA becomes active when helicases unwind the molecule, enabling promoter binding and the initiation of transcription or replication. (B) Secondary structures in RNAs arise from the hydrogen bonding between complementary base pairs within the molecule. Hairpin loops and other types of folding can reduce translational efficiency, but also protect the RNA from enzymatic degradation. (C) Protein activity is altered by phosphorylation events, where the addition of a phosphate group (red circle) to an amino acid changes the size, shape, or charge of a protein with consequences for function. (D) Enzymes can be deactivated by interactions with proteins or other molecules that bind to or obstruct the active site, preventing the enzyme from catalysing its reaction.

The first criterion for chemical dormancy is that a molecule must be able to exist in different states of activity. Sometimes, a molecule is considered ‘active’ or ‘inactive’ based solely on its concentration, since the reaction rate varies with the abundance of reactants in accordance with the rate law. However, this does not satisfy the first criterion of dormancy, which requires individual agents to occupy distinct states of activity. Instead, many biomolecules, including nucleic acids [16], vitamins [17] and proteins [18,19], exist in different states of activity depending on chemical reactions, environmental conditions and stochastic processes (figure 2). While in an active state, some molecules can catalyse reactions that promote their own synthesis. However, we do not consider autocatalysis or replication to be a defining criterion for chemical dormancy.

The second criterion for chemical dormancy is the ability of a molecule to reversibly transition between active and inactive states (figure 2). Such transitions can occur stochastically, driven by diffusion and random molecular encounters, which highlight the probabilistic nature of finite molecules within a specific volume. Examples include the formation of disulfide bonds between cysteine residues and other post-translational modifications, which can induce conformational changes that influence ligand binding and various aspects of protein function [18,19]. Transitions in chemical activity can also happen in a deterministic manner if initiated by changes in environmental conditions or through molecular interactions. For example, the activity of an mRNA strand is temperature-dependent. Portions of the molecule may form secondary structures at low temperatures, blocking translation, while higher temperatures cause these structures to melt, thereby allowing translation to proceed [16]. Taken together, these stochastic and deterministic transitions can affect reaction rates across molecules and contribute to the formation of complex, interacting and interdependent metabolic cycles.

The third criterion is that inactive molecules must be protected in ways that reduce the rates of decay from processes such as thermal degradation, photolysis, hydrolysis, or enzymatic reactions. For example, inside eukaryotic cells, DNA coils around histone proteins to form nucleosomes, the basic units of chromatin. This enhances molecular stability by increasing resistance to radiation and denaturing agents while also suppressessing gene expression and DNA repair [20] (figure 2A). Similar processes occur in archaea, where histone-like proteins facilitate DNA binding, thereby raising the DNA’s melting temperature and reducing the rate of radiolysis [21]. Dormancy-like processes also occur outside of cells. In soils, microorganisms release enzymes into their environment. Under dry conditions, these exoenzymes are protected via adsorption to minerals. Upon rewetting, these enzymes reactivate and resume catalysing biogeochemical reactions [22].

### (b) A test of chemical dormancy

**Figure 3.**
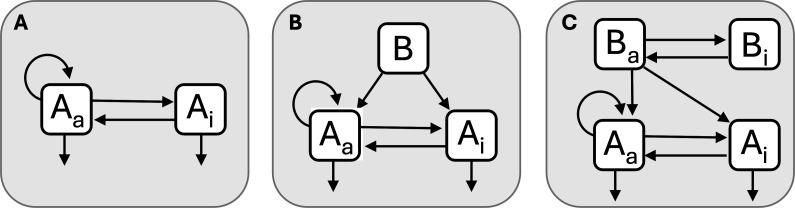
Chemical dormancy with a catalyst. **(**A) Molecule A only replicates when it is in an active state ($A_{a}$), but most individuals in the population exist in an inactive state ($A_{i}$). (B) Molecule B is a catalyst that binds to A, triggering its activation and leading to increased replication. (C) If B can also engage in dormancy, the activity and replication of A will be greater where and when B is active ($B_{a}$) as opposed to inactive ($B_{i}$). To meet the third criterion of chemical dormancy, we assume that inactive molecules are protected and thus decay more slowly than active molecules.

**Figure 4.**
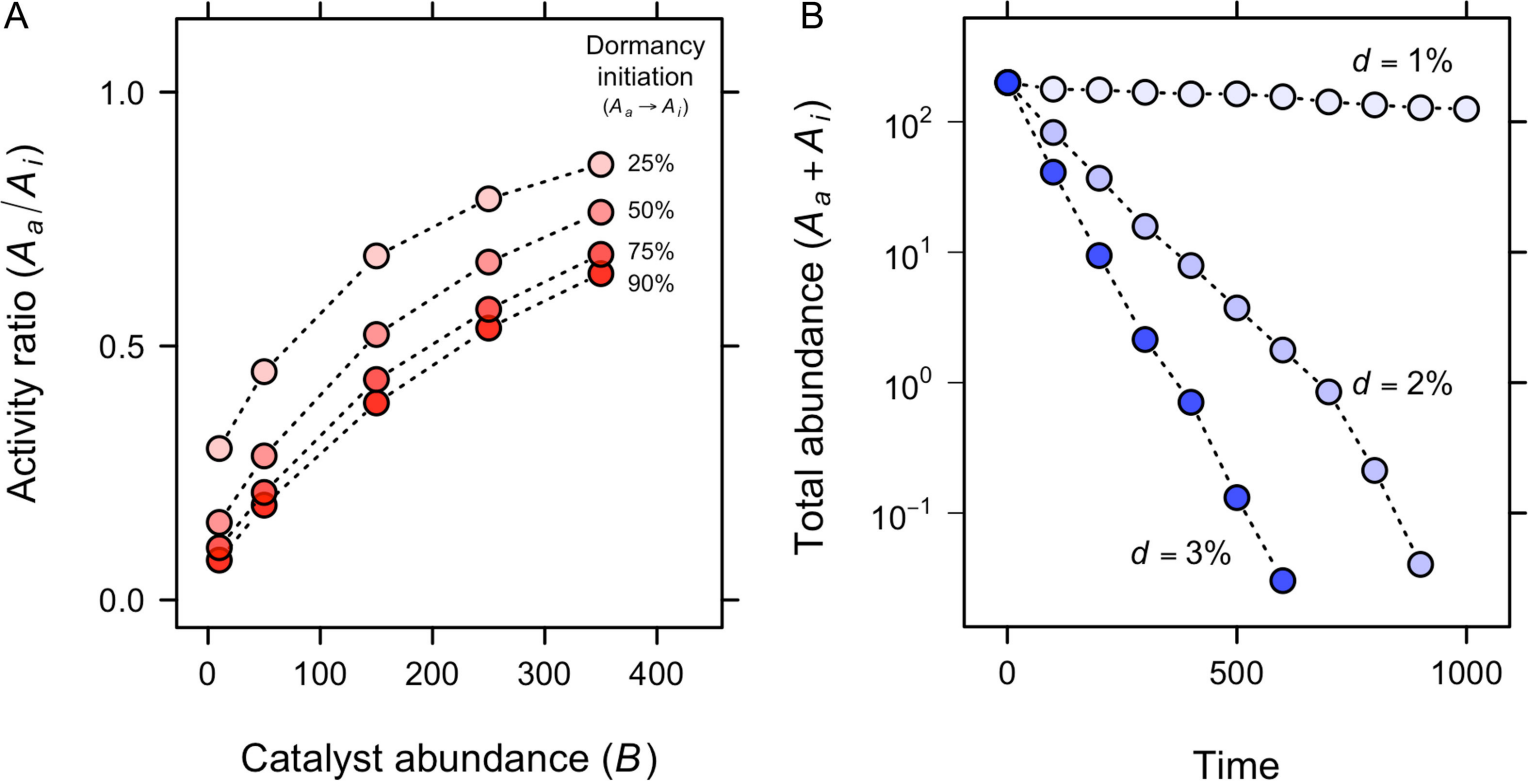
Autocatalytic simulations of chemical dormancy. (A) The ratio of active molecules ($A_{a}$) to inactive molecules ($A_{i}$) increases with catalyst (B) abundance in a simple autocatalytic simulation. (B) The total abundance of molecule A exponentially decreases with the rate that inactive molecules decay consistent with the view that protection afforded by dormancy can contribute to the persistence of molecules over time. Dormant molecules decay at each time step with probability *d*.

Since many biologically relevant chemicals meet the criteria for dormancy (figure 2), hypotheses can be formulated about how dormancy and molecular dynamics may have influenced the origin of life. Consider a prebiotic autocatalytic molecule, A, which exists mostly in an inactive state ($A_{i}$), but is capable of replication when in an active state ($A_{a}$). Another molecule, B, can bind to A and increase its replication rate. Over time, molecule A will accumulate in environments where molecule B is present. If B also has the ability to engage in dormancy, the synthesis of A will occur when and where B is active ($B_{a}$). In this scenario, dormancy modifies chemical interactions, shifting the optimal conditions for replication in ways that affect proliferation and persistence (figure 3). We used a simple individual-based model to explore these ideas where molecule A replicates and decays while stochastically transitioning between active ($A_{a}$) and inactive ($A_{i}$) states in the presence of catalyst B (figure 3B). We found that the proportion of active molecules ($A_{a}$) increases with catalyst (B) abundance (figure 4A). Furthermore, dormancy-mediated protection from decay increases the total abundance of A, making it less susceptible to molecular extinction (figure 4B).

In the autocatalytic model, molecule A has properties that are analogous to nucleic acids. DNA primarily exists in a dormant state, physically shielded from decay. The recruitment of promoters and polymerases facilitates the unwinding, activation and replication of DNA, paralleling behaviour of molecule B. Today, DNA is typically confined within cells. However, before the origin of life, nucleic acids likely formed in extracellular environments [23]. In such settings, RNA could bind to surfaces such as glass or montmorillonite, enhancing its stability and persistence [24,25]. Additionally, RNA takes on different secondary structures in response to temperature changes (figure 3), which affects its molecular activity and turnover [16]. Given the expansive mineral surfaces and fluctuating temperatures that were common on prebiotic Earth, nucleic acids probably underwent reversible changes in activity, consistent with the principles of chemical dormancy.

It is conceivable that dormancy-like properties contributed to the information-driven replication of life on Earth. Single-stranded nucleic acids experience higher rates of mutation than the more quiescent double-stranded nucleic acids. Simulations of polymer formation from monomers suggest the existence of a mutation threshold for self-replicating molecules [5]. When mutation rates exceed this threshold, the system fails to replicate with sufficient fidelity to sustain efficient self-copying. However, below this threshold, mutations are minimized enough to preserve functional integrity while still enabling genetic variation during replication, which is required for evolution by natural selection [5]. Thus, dormancy-like processes on early Earth may have mitigated ‘error catastrophe’—a potential challenge for polymers exposed to high mutation rates [26]—and facilitated the information-driven energy processing of life by altering molecular interactions.

## 4. Tracing the roots of dormancy in living systems

Dormancy is prevalent throughout the tree of life, with well-documented instances among extant lineages of viruses, bacteria, fungi, protists, worms, insects, crustaceans, amphibians, fish, birds, plants and mammals [10]. Despite its widespread occurrence, the genes and pathways that facilitate dormancy are not conserved across these diverse groups. Similar to traits such as vision, flight and echolocation, dormancy represents an example of homoplasy, more specifically, convergent evolution [27]. It has independently and repeatedly emerged across various lineages, suggesting that dormancy may represent a common solution to one of life’s universal challenges—living in a fluctuating and unpredictable environment. Here, we take a deep-time perspective to explore the distribution of dormancy throughout Earth’s history and investigate its potential origins.

### (a) Dormancy among ancient multicellular organisms

**Figure 5.**
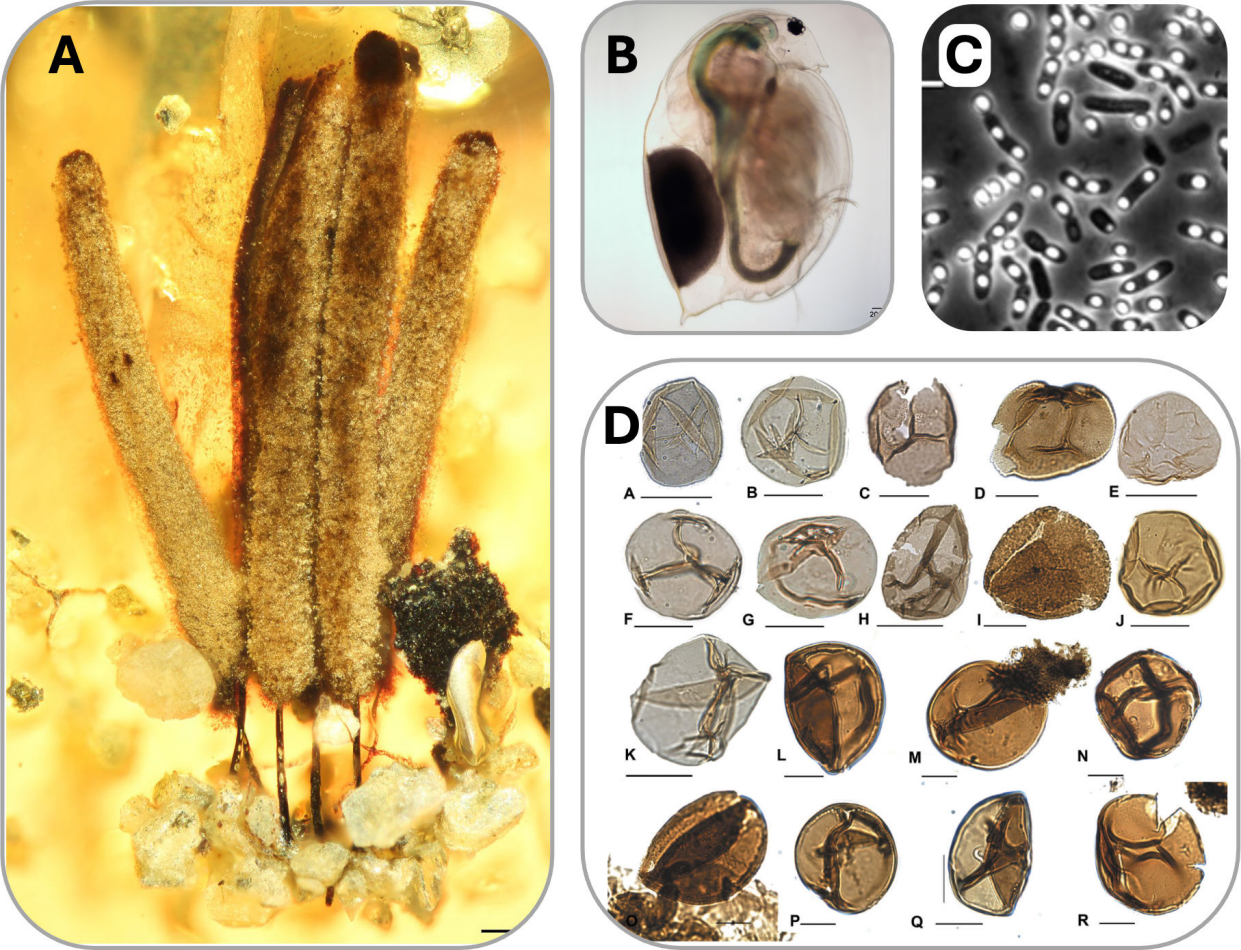
Examples of ancient dormancy. Dormancy is prevalent among extant lineages, but is also common in Earth’s fossil record. (A) Sporocarps of slime moulds preserved in amber 100 million years ago (Ma) [28]. (B) Resting eggs from aquatic crustaceans (*Daphnia*) have been recovered from 130 Ma sediments [29]. (C) Some bacteria related to *Bacillus* form long-lived endospores that have been resuscitated from permafrost dated to >1 Ma [30]. (D) Cryptospores related to primitive plants have been dated to 450 Ma [31]. Panel (B) image is courtesy of J. Haney and panel (C) image is courtesy of D. Schwartz.

**Figure 6.**
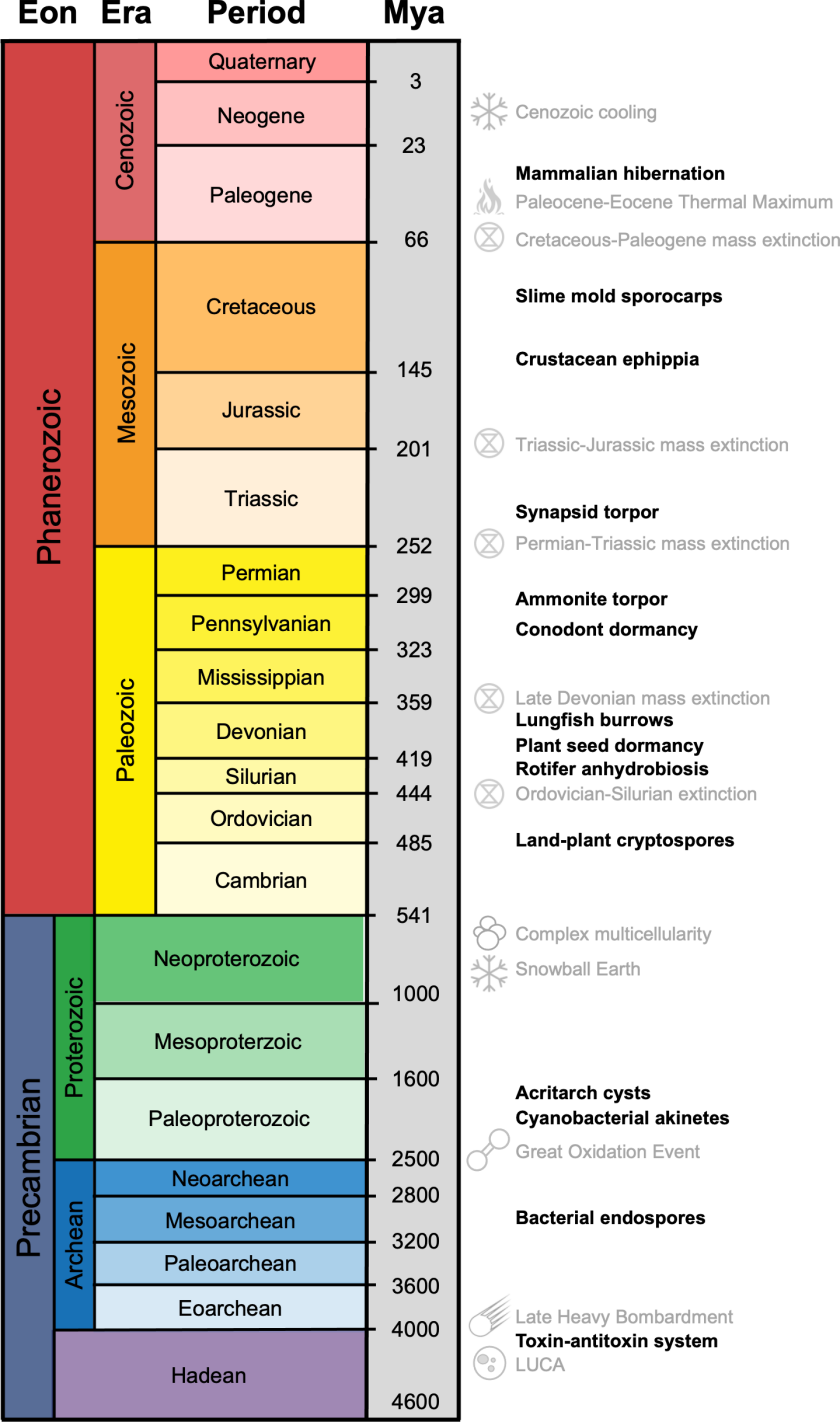
Dormancy through geological time. Grey text on the right margin highlights key biological and geological events throughout Earth’s history. Bold black text on the right margin corresponds with evidence of dormancy, either from the fossil record or from phylogenetic reconstructions.

Convergent evolutionary processes often point to the ancient origins of a phenotypic traits. Fossil evidence and phylogenetic reconstruction suggest that dormancy has been a long-standing strategy among plants, animals and microorganisms (figures 5 and 6). For example, slime mould sporocarps in amber dated to 100 million years ago (Ma) are consistent with the evolutionary stasis of dormancy-related morphological structures [28] (figure 5A). Resting stages produced by crustacean zooplankton (ephippia) have been recovered from 130 Ma lake sediments [29] (figure 5B). Evidence from the Early Triassic, around 250 Ma, suggests that a group of stem-mammals (synapsids) engaged in torpor to survive seasonal cycles at high latitudes, as indicated by growth marks on tusks [32]. Burrows used by lungfish for aestivation during prolonged periods of desiccation have been found in Devonian deposits [33]. Ancestral state reconstruction indicates that morphological and physiological aspects of dormancy had already emerged by 370 Ma among some of the earliest seed plants [34]. Dormancy may have even played a role in the transition of some phototrophs from water to land based on the recovery of cryptospores from 480 Ma [35].

### (b) Dormancy among ancient unicellular organisms

Dormancy existed among some major groups of microorganisms even before the Cambrian explosion. For example, fungus-like spores discovered in karst cavities dating to the Ediacaran period (635 Ma) led to speculation about the role of dormancy in population recovery following Snowball Earth events [36]. More compelling evidence of dormancy from the Proterozoic Eon comes from bacteria. Certain lineages of filamentous cyanobacteria (Nostocales and Stigonematales) produce akinetes (from Greek: ‘motionless’). These specialized resting structures are formed in response to environmental stressors, such as light or phosphorus limitation, and involve cell–cell signalling [37]. Akinetes are characterized by their reinforced cell walls, composed of a multilayered extracellular envelope. Their larger size accommodates the storage of carbon (glycogen) and nitrogen (cyanophycin), allowing akinetes to tolerate cold temperatures and desiccation for extended periods of time [37]. Remarkably, akinetes have been resuscitated from ocean sediments over 400 years old [38]. Although microfossils resembling modern filamentous cyanobacteria have been found in 3.5 Ba chert from Western Australia, the integration of phylogenetic, palaeontological and geochemical data suggests that akinetes evolved later, between 2.10 and 2.45 Ba [39].

Another ancient form of bacterial dormancy is endospore formation (figure 5C). It is estimated that modern-day marine subsurface sediments harbour approximately $10^{28}$ endospores [40]. Considering the total number of bacterial and archaeal cells on Earth is around $10^{29}$ [41], dormant endospores comprise at least 10% of the global microbial biosphere. Endospore formation is restricted to a few major lineages within Bacillota phylum. These bacteria make endospores in response to energy limitation and other environmental stressors. This well-studied mechanism of dormancy involves a complex development program that requires the regulation of more than 100 genes [42]. The resulting endospore is a water-depleted structure composed of over 70 different proteins [43]. Endospores can withstand extreme environmental conditions, including the vacuum of space [44] and simulated surface conditions on Mars [45]. As a result, endospores are capable of persisting for extended periods, as demonstrated by their revival from million-year-old permafrost samples [30].

Endospores are conservatively thought to have evolved at least as early as 3.0 Ba. This estimate is supported by two key pieces of evidence. First, comparative phylogenomics of ribosomal and sporulation proteins suggest that the last common ancestor of the Bacillota phylum was a spore-former [46]. Second, time-calibrated phylogenetic reconstructions place the divergence of Bacillota at approximately 2.9–3.1 Ba [47]. However, observations based on cell wall architecture have led to speculations that endosporulation could be much older. Most bacteria within the Bacillota are monoderms, meaning they have a single membrane as the outermost cellular structure. During sporulation, the mother cell engulfs the developing spore in a phagocytosis-like process, which continues until the spore matures and the mother cell is ultimately lysed. The ‘failed endospore origin’ hypothesis proposes that an incomplete final stage of endospore development led to the evolution of the first diderm bacteria, which are characterized by having two membranes [12]. An extension of this logic is that the last bacterial common ancestor (LBCA) was a spore-former [12], a claim that is consistent with network-based metabolic models [48]. While sporulation may have helped cells persist during harsh conditions that were characteristic of the early Earth, criticisms have been raised about the singularity of diderm origin [49]. Subsequent studies analysing a wide range of bacterial genomes have revealed that monoderms are not monophyletic [50], thereby weakening the claim that the LBCA was a spore-forming microorganism.

### (c) Easier forms of dormancy

Dormancy evolved early and often during Earth’s history. However, if it played a role in the emergence of life, early progenitor cells likely relied on simpler forms of dormancy. There are mechanisms by which microbial metabolism can be temporarily suspended without the need to produce cysts, resting stages or other specialized morphological structures. One way this can be achieved is with toxin–antitoxin (TA) systems. TA systems are diverse and can affect different processes, including plasmid retention and programmed cell death. TA systems can regulate cellular metabolism using only a pair of genes, making them a simpler but effective form of dormancy [51]. On a single operon, one gene encodes a protein that is the toxin, while the other gene encodes an RNA or protein that binds to and inactivates the toxin [52]. When a cell is physiologically stressed, antitoxins are degraded by induced proteases, which increase the concentration of unbound toxins in the cytosol. These free toxins can then inhibit processes such as DNA replication, mRNA stability, translation, cell wall biosynthesis and ATP synthesis, ultimately reducing microbial growth [51]. While the exact timing of the evolution of these systems remains uncertain, it is hypothesized that TA modules, or primitive versions of them, may have emerged shortly after the first cells evolved [53].

### (d) Potential origins of dormancy

The random processes occurring inside cells, which create variability in metabolic activity, may have contributed to the emergence of a primitive, dormancy-like phenotype. Gene expression varies among individual cells owing to stochastic encounters between transcription factors and promoter sequences, a process that is critical for initiating transcription by a relevant RNA polymerase [54]. Similarly, protein translation occurs in bursts driven by the stochastic binding of ribosomes to mRNA [55], while signal transduction is noisy due to the inherent randomness of ligand-receptor binding [56]. Cellular activity is also influenced by epigenetic processes where methyl groups randomly bind to nucleotides, which can silence genes essential for metabolic functioning [57]. The stochasticity of these processes generates heterogeneity within a population. Even when isogenic cells are maintained under nearly identical conditions, metabolic activity often exhibits a long-tail distribution [58], where a small proportion of cells remain highly active, while the majority exist in a reduced metabolic state.

Variation in metabolic activity among individuals is an inherent and unavoidable feature of life. The discrete and probabilistic nature of molecular interactions—such as collisions, binding events and diffusion—is fundamental for the functioning of cells today, but also for life processes on early Earth. This leads us to hypothesize about the origins of dormancy. We propose that variation in metabolic activity among individuals in a population of progenitor cells would inevitably be generated by numerous random multiplicative processes. In a fluctuating environment, some of these primitive organisms would survive longer by conserving energy and tolerating stress during periods unfavourable for growth and reproduction. These individuals could then resume activity when conditions improved, not only contributing to the persistence of life but also becoming subject to natural selection. Over time, this process would lead to refinement, ultimately giving rise to more complex forms of dormancy that were finely tuned to Earth’s conditions that were becoming increasingly stable and conducive to life.

## 5. Consequences of dormancy on early Earth

Dormancy is a life-history strategy that has shaped the evolution of contemporary life on Earth. Given its ancient origins (figures 5 and 6), dormancy likely played an important role in the past, potentially contributing to the emergence and persistence of life on early Earth. Although conditions around 4 Ba were vastly different from those of today, the fundamental rules of biology remain the same. Primitive organisms still would have needed to acquire energy, resist entropy and behave according to the basic principles of ecology. From this perspective, we consider how dormancy might have affected early life.

### (a) Dormancy buffering of small populations

One difference between the early and modern biosphere is the overall abundance of life. Population sizes were likely much smaller, rendering them more vulnerable to stochastic events, with implications for demography and evolution. In small populations, genetic drift outweighs selection, preventing beneficial mutations from increasing in frequency and allowing mildly deleterious mutations to persist [59]. Consequently, maladaptive alleles can accumulate, decreasing fitness and potentially driving populations to extinction through a phenomenon known as Muller’s ratchet [60]. In addition to lowering the input of mutations associated with genome replication, dormancy also reduces the influence of genetic drift by increasing the effective population size [61], which may have contributed to the maintenance of genetic diversity on early Earth.

### (b) Dormancy-mediated dispersal

Today, it is estimated that there are upwards of $10^{12}$ species on the planet, a result of the ongoing balance between speciation and extinction over the past 4 billion years [62]. In contrast, the early biosphere contained far fewer species and many more vacant niches. A major challenge for primitive organisms would have been successfully dispersing to these niches. If life first emerged in the oceans, it may have had access to abundant resources and been able to more easily disperse while remaining in a metabolically active state [63,64]. Alternatively, if life first emerged in inland volcanic hot springs [65,66], the early Earth could be envisioned as a mosaic of sparsely distributed habitable patches. In this scenario, dormancy could increase survival during transit and facilitate colonization of new environments [67] (figure 1). Upon reaching an unoccupied site with minimal competition, resuscitation and growth could lead to local establishment and subsequent range expansion. Moreover, by broadening the parameter space for evolutionary branching and promoting genetic divergence between subpopulations, dormancy may have played a key role in facilitating adaptive radiations [68].

### (c) Dormancy protection against disturbance

Life first emerged on Earth during a tumultuous time. Throughout the Hadean Eon (4.6–4.0 Ba), meteorites repeatedly struck the planet, melting Earth’s crust and potentially driving nascent life to extinction [69]. Throughout most of the Eoarchean era (4.0–3.6 Ba), during the late heavy bombardment, large impactors—some exceeding 50 km in diameter—may have struck Earth approximately once every million years [70]. Although these collisions were probably too infrequent to directly select for dormancy in early microbial populations, dormancy may have contributed to post-impact survival as well as facilitated reseeding events from ejecta [71]. In the unlikely event of surface sterilization, life may have survived within igneous rocks [70], forming a subsurface refugium of dormant microorganisms [72].

### (d) Dormancy defence against viruses

One feature of the early biosphere that is consistent with today’s biosphere is that life had to contend with infection from viruses. Many viruses are thought to have already been present at the time of the last universal common ancestor (LUCA) [73]. Reconstructions suggest that LUCA was an anaerobic acetogen that likely possessed a viral immune system, as inferred from the conservation of more than a dozen CRISPR–Cas effector proteins [74]. Some Cas nucleases induce dormancy in bacterial hosts, thereby clearing viral infections and providing herd immunity to uninfected cells [75]. Other forms of dormancy, such as endosporulation and persister cell formation, can inhibit phage infections [76,77]. There are even reports suggesting that mere physical contact with virus particles can trigger cellular inactivity [78]. Collectively, these observations suggest that dormancy may represent a long-standing defence mechanism against the pressures of cellular infection.

## 6. Astrobiology and dormancy

A major goal of astrobiology is to determine whether life exists elsewhere in the universe. Mars has long been of significant interest, not only because of its proximity to Earth but also due to surface features shaped by water [79]. Similarly, some icy moons of Saturn and Jupiter, like Enceladus and Europa, have liquid water beneath their frozen surfaces. However, life on these planetary bodies would face challenges from multiple stressors, including (but not limited to) high pressures, oscillating temperatures, and limited access to nutrients and energy. It is plausible that extraterrestrial life might possess dormancy-like capacities to survive the variability and extreme conditions driven by climatic and planetary processes within our solar system. Dormancy has been hypothesized to be an adaptation for potential life on Mars, given the radical changes that have occurred throughout its geologic history [80]. In the search for life within our solar system, astrobiologists have identified biosignatures that may indicate past or present life. These include information related to atmospheric gases, isotopic fractionation of key elements, fossilized remains and organic compounds (e.g. isoprenoids), as well as microbially induced sedimentary structures [81,82]. Additionally, dormancy-like phenotypes should be considered as potential biosignatures. If extraterrestrial life exhibits any similarities to life on Earth, it is possible dormancy could be inferred through nucleic acid ratios (RNA : DNA), resuscitation assays, or the detection of cysts, resting cells and spores [8,80].

Outside of our solar system, interest in life detection has been motivated by the deployment of advanced telescopes that have led to the discovery of previously unknown exoplanets. To date, nearly 10 000 confirmed and candidate exoplanets have been identified [83], with a small fraction residing in habitable zones based on criteria such as size, composition, orbit, stellar luminosity, proximity to the host star and the existence of a water cycle [84]. A major challenge in studying the habitability of exoplanets is their vast distances from Earth, making direct study intractable. Consequently, the search for life on exoplanets often focuses on detecting atmospheric redox disequilibrium through interferometry and spectrometry [81]. Distinguishing a lifeless planet from one with a predominantly dormant biosphere could be difficult using atmospheric signatures alone, as the reduced signal-to-noise ratio relative to the background complicates detection. However, life might still be inferable if it has altered the planet’s surface, especially by increasing the complexity and diversity of the planet’s mineral assemblage. It is estimated that 34% of the minerals on Earth’s surface are only formed by biology [85]. Thus, a planet with a dormant yet persistent biosphere could potentially be identified via reflectance spectrometry of surface minerals.

## 7. Looking forward

Understanding the emergence of life requires insights from biology, geology, chemistry, astronomy and physics. Despite significant advancements across these disciplines, reconstructing Earth’s early history is difficult, largely due to the loss of material and information over vast expanses of time. Despite this, origins-of-life research has developed rigorous approaches to ensure that strong inferences can be made. For example, we demonstrate how the fossil record and phylogenetic reconstructions can be used to describe the distribution and timing of dormancy-related processes in Earth’s past (figure 5).

### (a) Opportunities for metagenomic insights

Metagenomics is a field that has revolutionized our ability to assemble and analyse the genomes of bacteria, archaea, eukaryotes and viruses from virtually all habitats on the planet, including the deep subsurface and other extreme environments that may resemble conditions where life first evolved. These efforts have led to the discovery of numerous deep-branching lineages, shedding light on long-standing debates regarding the evolutionary relationships between the major domains of life [86]. Moreover, metagenomic approaches have facilitated the development of genome-scale models that can generate hypotheses relating to the metabolism and traits of LUCA [74].

In the context of dormancy, metagenomics has helped identify candidate bacterial phyla, like the Dormibacteraeota. As the name suggests, these microbes possess genes that are likely associated with dormancy, including those involved in the synthesis and degradation of glycogen, a storage molecule that may support maintenance energy costs, thus extending the duration of cellular viability in an inactive state [87]. Surprisingly, Dormibacteraeota also harbour a significant number of endosporulation genes [87], although not enough to meet the minimal requirement for producing a functional endospore. These observations raise intriguing new questions about the origins and distribution of ancient forms of dormancy.

### (b) Opportunities for modelling insights

Modelling approaches are essential for formalizing and testing hypotheses about the emergence of life on early Earth. For example, self-sustaining chemical reaction networks, chain formation and self-replication necessary for the evolution of life have been evaluated using autocatalytic sets and kinetic models [88,89] (figures 3 and 4). In addition, panspermia is the hypothesis that life exists throughout the universe and can be transported among celestial bodies. Analytical modelling has explored how debris generated from meteorite collisions could exceed planetary escape velocities and potentially spread life throughout the Milky Way [90]. Organisms embedded in ejecta (e.g. dust, rocks or ice) would be subjected to harsh conditions, including extreme temperatures, elevated exposure to high-energy particle radiation, gamma-ray bursts and cosmic rays. For this reason, survival parameters that reflect protection from dormancy are included in models used to make quantitative predictions about panspermia [90].

Dormancy has also been incorporated into models of protocell evolution. These models couple growth–division dynamics to chemical reactions, which allow individuals to spontaneously transition between active and inactive states [91]. In a population of protocells with heritable variation, active cell types evolve to become dominant, while inactive types persist at a lower frequency [91]. Dormancy has also been incorporated into ‘game of life’ models, where extinction dynamics are shaped by individuals transitioning between metabolic states based on the density of neighbouring individuals and the stochastic survival of spores [9]. These types of models have the potential to identify what is minimally required for the emergence of complex behaviours associated with dormancy, which is relevant to origins-of-life research.

### (c) Opportunities for experimental insights

Experimental approaches offer promising avenues for uncovering how dormancy shapes the evolution of chemical and biological systems. For example, predictions derived from autocatalytic networks (figures 3 and 4) can be tested in the laboratory by subjecting molecules of varying types and sizes to mutation and selection, thereby evaluating their potential for replication, self-organization and evolution [92]. Such an experimental platform could be extended to investigate chemical dormancy, which would require characterizing the distribution of molecular activity under fluctuating environmental conditions. Integrating such studies with quantitative models would offer a novel and rigorous means of testing whether dormancy-like processes may have contributed to the stability of chemical precursors required for the origin of life.

Experiments can also provide valuable insight into the role of dormancy in the evolution of living systems. Experimental evolution involves the tracking of genotypic and phenotypic changes in replicate microbial populations over time in response to environmental conditions. This approach was used to understand how the loss of dormancy affected the molecular evolutionary dynamics of *Bacillus* exposed to long-term bouts of feast and famine [93] (figure 5). A palaeo-molecular framework can also be used to explore the evolution of traits across geological time scales. This involves reconstructing ancestral sequences, which are then synthesized and cloned into modern-day organisms [94]. Through this resurrection process, it is possible to gain insight into the conservation and adaptation of proteins over time. When integrated with theory, experimental approaches are poised to yield significant breakthroughs in understanding the evolution of ancient forms of dormancy (figure 6).

## 8. Conclusions

Many living and non-living systems exhibit dormancy-like properties, enabling agents to transition between states of activity either stochastically or in response to environmental conditions. The protection afforded by dormancy allows inactive agents to persist during suboptimal conditions and resume activity when more favourable conditions return. These features of dormancy create a ‘seed bank’ that buffers population dynamics and preserves information, which can lead to the emergence of memory, complexity and feedback. Widespread among diverse lineages today, dormancy was prevalent among various forms of life in the distant past, likely present at the time of LUCA, perhaps even earlier. Dormancy could have shielded life from extinctions, facilitated dispersal, reduced competition and enhanced diversification during the development of the early biosphere. By acting on biomolecules in a prebiotic world, dormancy-like properties may have even played a role in the origin of life. The criteria for dormancy are relatively simple and appear easy to evolve, suggesting that dormancy is a general, if not universal, solution to one of life’s most fundamental challenges: persisting in fluctuating and unpredictable environments.

## Data Availability

All data and code are available via Zenodo [95].
